# Regulatory Effects of *FGF9* on Dermal Papilla Cell Proliferation in Small-Tailed Han Sheep

**DOI:** 10.3390/genes14051106

**Published:** 2023-05-18

**Authors:** Qi Jia, Shuangshuang Zhang, Dan Wang, Jianqiang Liu, Xinhui Luo, Yu Liu, Xin Li, Fuliang Sun, Guangjun Xia, Lichun Zhang

**Affiliations:** 1 Institute of Animal Biotechnology, Jilin Academy of Agricultural Sciences, Gongzhuling 136100, China; 2 College of Agriculture, Yanbian University, Yanji 130021, China

**Keywords:** fibroblast growth factor 9, small-tailed Han sheep, dermal papilla cells, proliferation

## Abstract

Fibroblast growth factor 9 (*FGF9*) is crucial for the growth and development of hair follicles (HFs); however, its role in sheep wool growth is unknown. Here, we clarified the role of *FGF9* in HF growth in the small-tailed Han sheep by quantifying *FGF9* expression in skin tissue sections collected at different periods. Moreover, we evaluated the effects of *FGF9* protein supplementation on hair shaft growth in vitro and *FGF9* knockdown on cultured dermal papilla cells (DPCs). The relationship between *FGF9* and the Wnt/β-catenin signaling pathway was examined, and the underlying mechanisms of *FGF9*-mediated DPC proliferation were investigated. The results show that *FGF9* expression varies throughout the HF cycle and participates in wool growth. The proliferation rate and cell cycle of *FGF9*-treated DPCs substantially increase compared to that of the control group, and the mRNA and protein expression of *CTNNB1*, a Wnt/β-catenin signaling pathway marker gene, is considerably lower than that in the control group. The opposite occurs in *FGF9*-knockdown DPCs. Moreover, other signaling pathways are enriched in the *FGF9*-treated group. In conclusion, *FGF9* accelerates the proliferation and cell cycle of DPCs and may regulate HF growth and development through the Wnt/β-catenin signaling pathway.

## 1. Introduction

Small-tailed Han sheep have bright coloured hair with a fine and soft texture, making it a good raw material for leather. However, compared with that of Merinos, its wool production performance needs to be improved [1]. Wool is an essential production material and is one of the most economically important traits of sheep. HFs form the basis of wool development, and their physiological activities determine the growth and physical properties of their wool product. The density, fineness, and curvature of hair are primarily determined by HF function. HFs are the only mammalian mini-organs with a lifelong periodic cycle that regenerate by spontaneously undergoing repeated anagen, catagen, and telogen phases [2]. Although HFs are highly regulated by various growth factors, cytokines, neuropeptides, and hormones, the periodic HF cycle is a self-sustaining phenomenon that can persist even when HFs are isolated from organ cultures. Despite the identification of multiple molecular regulators in mutant mouse models with defective HF cycles and characteristic gene expression patterns in different phases, the molecular mechanisms that drive the HF cycle remain unclear [3].

DP, which are found at the base of the HF, serve as regulatory centres and are essential for the formation, development, and regulation of HFs and their cycles [4,5]. They are crucial for morphogenesis and controlling the periodic regeneration of HFs. Only DPCs constitute the DP and are mainly distributed at the base, encircled by hair matrix cells [4]. Numerous growth factors and intercellular signaling molecules, such as IGF-1, KGF, and HGF, are expressed and secreted by DPCs and act on neighbouring cells to control cell proliferation and differentiation in HFs, as well as hair shaft elongation [5]. When in vitro DPC cultures were transplanted into nude mice, the mice grew many HFs with visible hair; if DPCs were reduced, HFs failed to form complete structures [6,7,8]. Dermal papilla cells can also regulate the size and length of hair shafts by inducing the growth and differentiation of hair matrix cells [9]. In vitro cultures show that DP can also aid in HF regeneration. Moreover, DPCs do not only induce epithelial cells to form HFs during the embryonic period, but also regulate the periodic regeneration of mature HFs after birth [10,11]. Signaling pathways and cytokines that regulate the developmental and biological characteristics of the DP are expressed in DPCs, and the number and type of DPCs can determine the size and shape of the hair [11,12]. Research on DP is crucial to understand HF formation and hair growth; therefore, we conducted experiments with DPCs at the core.

The FGF gene family, which is widely expressed in skin HF tissues, plays an important role in the cell proliferation, differentiation, and the periodic growth cycle of HFs. It also participates in many other biological processes in mammals by binding to FGFR and cross-synergising with signaling pathways such as neurogenic NOTCH, WNT, and TGF-β [13,14,15]. Kawano et al. conducted a quantitative analysis of the mRNA expression of 22 FGF family members and four FGFRs in the skin of adult mice at different stages of the hair growth cycle [16]. They found that *FGF1*, *FGF2*, *FGF5*, *FGF7*, *FGF10*, *FGF13*, *FGF18*, and *FGF22* were expressed at different stages of the HF proliferation cycle and that their expression peaks varied. The mRNA expression of *FGF18* and *FGF13* peaked during the telogen phase, that of *FGF7* and *FGF10* peaked during anagen phase V, and that of *FGF5* and *FGF22* peaked during anagen phase VI. Housley et al. demonstrate that *FGF5* inhibits hair growth by promoting the transition from the anagen to the catagen phase in HFs [17]. *FGF9* participates in promoting the formation of dermal agglutinates during the early developmental stage of HFs [18,19]. Studies show that human peripheral blood γ delta (γδ) T cells can express *FGF9* in response to stimulation by isopentenyl pyrophosphate and TGF-β1/interleukin (IL)-15, play important roles in skin tissue injury recovery, and indirectly promote the regeneration of HFs [20]. Komi-Kuramochi et al. [21] and Zheng et al. [22] reported similar results regarding the crucial roles of *FGF9* in wound healing.

In vitro and in vivo studies suggest that numerous cytokines of the FGF family have regulatory effects on the growth and development of HFs, among which mutations in *FGF5* can maintain a prolonged anagen phase in the HFs of multiple species, resulting in a long-hair phenotype [23]. *FGF5* gene-edited sheep produced using CRISPR/Cas9 technology by Zhang et al. showed a significant increase in hair length and average growth rate [24]. Moreover, the *FGF21* gene regulates the proliferation and differentiation of fibroblasts and the formation of HFs during the first growth cycle by promoting the transition from the anagen to the catagen phase, acting mainly during the anagen phase [25]. Kimura-Ueki et al. [26] found that *FGF18* is expressed in HF stem cells throughout the telogen phase by conditionally knocking out the *FGF18* gene in keratin-5-positive epithelial cells of genetically engineered mice, revealing that *FGF18* regulates the hair cycle mainly through the non-growth phase. Shu et al. [27] showed that during their first growth cycle, *FGF7* and *FGF10* might induce HFs to enter a new cycle, whereas *FGF22* might play an important role in inducing HFs to enter the catagen phase. However, studies also show that *FGF7* is necessary for hair growth but not wound healing, whereas FGF10 is necessary for embryonic epidermal morphogenesis but not development [28,29]. Therefore, members of the FGF family act differently during different HF growth stages and maintain normal HF growth and development. However, there is no direct evidence that *FGF9* regulates the growth of sheep wool. The *FGF9* gene might participate in determining the composition of sheep HF traits; however, its exact regulatory mechanism remains unclear.

Due to the crucial roles of both DPCs and *FGF9*, we designed this study to gain an overview of the cellular functions of *FGF9* in DPCs and further elucidate the roles of *FGF9* in HF growth and development. Therefore, we hypothesised that *FGF9* might participate in HF growth and development by regulating cell processes in DPCs. We explored the effects of *FGF9* on wool growth by culturing HFs of small-tailed Han sheep in vitro and examining the changes in *FGF9* gene expression. To speculate the potential functions of *FGF9* as a candidate gene for the growth and development of HFs, we further investigated the roles of *FGF9* by examining the effects of additional *FGF9* protein treatment and *FGF9* knockdown on the proliferation and cell cycle of DPCs. Lastly, transcriptome analysis was performed to gain new insights into the molecular mechanisms through which *FGF9* affects the function of DPCs. This study provides a scientific basis for subsequent studies on wool growth and development in sheep, thereby laying the foundation for exploring the genetic mechanisms underlying the composition of different wool traits.

## 2. Materials and Methods

### 2.1. Sample Collection

Three two-year-old female small-tailed Han sheep were used in this study. These sheep have no oestrus, pregnancy, or lactation period, and their wool production, stretch length, and curl are all lower than Merino sheep. Therefore, there remains room for improvement in wool production performance [1]. Their outer layer of wool was removed by localised shearing of the scapulae, whereby shears were applied close to their dorsal lateral skin. The skin was cleaned of any remaining fleece. Then, the skin was sterilised by wiping with 75% alcohol, and a 0.5 cm^2^ sample of skin tissue containing different HF cycles was collected using sterile surgical shears. Prior to sample collection, the sheep were locally anaesthetised using brucine hydrochloride. The sheep were raised by the Jilin Academy of Agricultural Sciences. Skin samples were collected in January, April, and July of 2022. The research protocol was reviewed and approved by the Animal Welfare and Ethics Committee of Jilin Academy of Agricultural Sciences (AWEC2021A03, 28 May 2021).

### 2.2. Sectioning and Preparation of Skin Tissues

Fresh skin tissue samples were trimmed, fixed in 4% paraformaldehyde (Boster, Wuhan, China) for 48 h, and rinsed in running water for 24 h. After dehydration via immersion in 70%, 75%, 85%, 95%, and 100% alcohol for 1 h each, the samples were soaked in xylene for 20 min and then in wax for approximately 6 h for embedding. The samples were then longitudinally cut in the direction of the HFs, stained with haematoxylin and eosin (HE; Dingguo, Shanghai, China), and sealed with neutral resin. The tissues used for RNA extraction were immersed according to the manufacturer’s instructions in RNAlater preservation solution (Thermo Fisher Scientific, Waltham, MA, USA), and the tissues used for DPC extraction were immediately sent to the laboratory (Institute of Animal Biotechnology, Jilin Academy of Agricultural Sciences, Gongzhuling, Jilin, China) for cell isolation and culture.

### 2.3. Isolation and Culture of DPCs

DPCs were isolated using the methods described by Kobayashi et al. [30] and Topouzi et al. [31], which involved blunt dissection combined with enzymatic digestion. Briefly, the scapular skin of small-tailed Han sheep was digested with neutral protease at 4 °C for 4 h. HFs in the anagen phase with intact DP were selected, and the top of the hair bulbs were cut off under a microscope (Nikon eclipse Ti-s, Tokyo, Japan; 100× objective). The DPCs were then removed and transferred to a Dulbecco’s modified Eagle medium/nutrient mixture F-12 (DMEM/F12) (Gibco, Grand Island, NY, USA) medium with 10% fetal bovine serum (FBS) (Sigma, St. Louis, MO, USA) and 1× antibiotic and antifungal agents (Sangon Biotech, Shanghai, China) using mouth pipetting. All cells were cultured at a constant temperature of 37 °C with 5% carbon dioxide (CO_2_) for approximately 7 days.

### 2.4. Exogenous Addition of FGF9 Protein and Inhibition of FGF9 Gene Expression

Recombinant human *FGF9* (Takara Bio, Beijing, China) was dissolved in sterile PBS containing 0.1% BSA according to the manufacturer’s instructions to prepare a stock solution at a concentration of 100 μg/mL. Thereafter, it was diluted to the target concentrations in DMEM/F12 (Gibco) containing 10% FBS (Sigma), according to the different dose requirements.

Three siRNA sequences, siRNA-*FGF9*-1, siRNA-*FGF9*-2, and siRNA-*FGF9*-3 were designed according to the mRNA sequence of the sheep *FGF21* gene published in GenBank (accession number: XM_027977590.1) and transfected into the DPCs using Lipofectamine 3000 (Thermo Fisher Scientific). The total RNA of the cells was extracted 48 h after transfection, and the transfection efficiency was tested using real-time fluorescence-based qPCR to screen for the best siRNA–*FGF9* combination for subsequent experiments. All siRNA sequences were constructed by Jintuosi Biotechnology Co., Ltd., Wuhan, China, and their sequences are listed in Table 1.

### 2.5. Effects of FGF9 on HF Growth In Vitro

HFs were isolated using the method described by Higgins et al. [22] and cultured in vitro under the following conditions: William’s E serum-free culture medium (Gibco) with 2 mmol/L L-glutamine (Sangon Biotech), 2 mmol/L HEPES (Sigma), 10 µg/mL insulin (Sigma), 10 ng/mL sodium selenite (Aikeshiji, Chengdu, China), 10 ng/mL hydrocortisone (Sigma), 10 µg/mL transferrin (Aladdin, Shanghai, China), 100 U/mL penicillin (Sangon Biotech), and 100 µg/mL streptomycin (Sangon Biotech) at 31 °C with 5% CO_2_ and saturated humidity. The treated HF group was additionally administered with 100 ng/mL *FGF9*. Eight intact HFs in the anagen phase were extracted from each group and placed in 24-well plates for in vitro culture. The HF length (the total height of the HFs containing new fibres minus the initial height) was measured daily (in millimetres), and the growth rate was plotted using GraphPad Prism software (version 7.0, GraphPad Prism software, Inc., La Jolla, CA, USA).

### 2.6. Determination of Cell Proliferation Rate and Cell Cycle

The isolated and cultured DPCs of small-tailed Han sheep were uniformly inoculated into 96-well plates at 100 μL/well (containing approximately 2000 cells). The original culture medium was then replaced with complete culture medium containing 0, 10, 100, and 1000 ng/mL *FGF9* protein after 24 h of inoculation. After 24, 48, 72, and 96 h, 10 μL CCK-8 solution was added to each well. The absorbance of each well at 450 nm was measured using a microplate reader after 2 h of incubation at 37 °C, and cell proliferation curves were plotted through GraphPad Prism 7.0 software.

The subsequent experimental conditions were set as 100 ng/mL *FGF9* treatment for 48 h. To further examine the facilitatory effect of *FGF9* on DPC proliferation, we evaluated the changes in the number of DPCs in the proliferative phase using a 5-ethynyl-2 deoxyuridine (EdU) assay. After exogenous addition of *FGF9* protein and transfection, DPC proliferation was evaluated using a Cell-Light EdU Apollo567 In Vitro Kit (RiboBio, Guangzhou, China), according to the manufacturer’s instructions. At 48 h after transfection, the cell cycle of the DPCs was examined using flow cytometry with the Cell Cycle and Apoptosis Analysis Kit (Beyotime, Shanghai, China) according to the manufacturer’s recommendations.

### 2.7. RNA Extraction, Library Construction, and High-Throughput Sequencing

A 1 mL volume of TRIzol (Invitrogen) was added to each treatment group. The bottom cells were repeatedly aspirated to form a cell suspension, which was transferred to a centrifuge tube. Then, 200 μL chloroform was added, and the tube was shaken to obtain a pink turbid solution. The solution was allowed to rest for 10 min at 4 °C before centrifugation for 15 min at 12,000× *g*. The upper colourless, aqueous phase was transferred to another centrifuge tube. Isopropanol (500 μL) was added, and the solution was mixed thoroughly, left to rest for 10 min at 4 °C, and then centrifuged at 12,000× *g* for 10 min. The supernatant was removed and discarded. Next, 1 mL 75% ethanol was added, and the tube was then shaken to resuspend the RNA precipitate at 4 °C. The suspension was centrifuged at 7500× *g* for 5 min, after which the supernatant was discarded and the precipitate was dried at room temperature in an ultra-clean table. Next, 20 μL diethyl pyrocarbonate water was added to fully dissolve the precipitate to obtain the RNA samples. The first strand cDNA was prepared using a PrimeScript™ RT Reagent Kit with gDNA Eraser (Takara Bio) according to the manufacturer’s instructions. The RNAs and cDNAs were stored at −80 °C and −20 °C, respectively, for later use. The quality of the RNA was measured using a Quawell Q5000 spectrophotometer (Quawell, San Jose, CA, USA) and RNA integrity was confirmed through agarose gel electrophoresis. The mRNAs were enriched using magnetic beads containing oligo-deoxythymidine (dT) and broken into short fragments by adding a fragmentation buffer. Subsequently, double-stranded cDNA was synthesised via reverse transcription using random hexamer primers. AMPure XP (Beckman Coulter, Beverly, MA, USA) beads were used to purify the double-stranded cDNAs and select fragments based on size, which were then amplified using PCR to construct a cDNA library. RNA was quantified using Agilent 2100 Bioanalyzer (Agilent Technologies, Santa Clara, CA, USA). After the library passed the quality control analysis, transcriptional sequencing was performed using an Illumina HiSeqTM2500/4000 sequencing platform (Allwegene, Beijing, China).

### 2.8. Bioinformatics Analysis

Raw sequencing data were filtered using Trimmomatic software (version 0.33). Cleaned data were mapped onto the reference genome of *Ovis aries* using STRA software (Reference Genome Version ARS-UI_Ramb_version 2.0 [NCBI]). Gene expression in each sample was analysed using the HTSeq software (version 0.5.4) in combination with a union model. The fragments per kilobase of exon model per million mapped reads (FPKM) value was used as a measurement of the gene expression level. A ∣log_2_ (fold change)∣ > 1 and *p* < 0.05 were used as the criteria to identify differentially expressed genes (DEGs).

### 2.9. qPCR

Differences in the expression of *FGF9* in skin tissues containing HFs in the anagen, catagen, and telogen phases were also tested using qPCR. *ACTB* was used as the internal control. After the exogenous addition of *FGF9* protein and inhibition of *FGF9* gene expression in DPCs, several key genes of the Wnt/β-catenin signalling pathway, including *CTNNB1*, *GSK3β*, *LEF1*, *DVL2*, *LRP5*, and *LRP6*, were selected for qPCR quantification. The first strand cDNAs were created after the total RNA was extracted. Using the Primer Premier (version 5.0) program, primers were created following the assembly sequences of the investigated genes in GenBank (Table 2).

### 2.10. Western Blot

After the DPCs were treated for 48 h, the proteins were collected with radioimmunoprecipitation assay buffer (RIPA) (Beyotime, Shanghai, China), a lysis buffer. Protein concentrations were measured using an Enhanced BCA Protein Assay Kit (Beyotime) according to the manufacturer’s instructions. Proteins were separated through sodium dodecyl sulphate–polyacrylamide gel electrophoresis (SDS-PAGE), transferred to PVDF membranes, and treated with 1:1000 rabbit anti-β catenin (Abcam, Cambridge, UK), and 1:2000 rabbit anti-glyceraldehyde 3-phosphate dehydrogenase (GAPDH) (Proteintech Group, Rosemont, IL, USA) antibodies, followed by 1:3000 goat anti-rabbit immunoglobulin G (IgG) horse radish peroxidase (HRP)-labelled antibody (Bioss, Beijing, China). The signals were detected using an ECL Western Blot Kit (Pripril, Beijing, China) according to the manufacturer’s instructions. Protein testing and analysis were performed using a ChemiScope 6000 Touch Imaging System (Clinx Science Instruments, Shanghai, China).

### 2.11. Gene Ontology (GO) and Kyoto Encyclopedia of Genes and Genomes (KEGG) Pathway Enrichment Analyses

To gain insight into the biological relationship between the DEGs of DPCs in the *FGF9* protein-supplemented and control groups, we set the significance threshold (*p* < 0.05) and used cluster analysis software to perform GO (GOSeq, topGO, v1.22) and KEGG (KOBAS, v2.0) pathway enrichment analyses on the DEGs. GO was divided into three categories: molecular function, biological processes, and cellular composition.

### 2.12. Statistical Analysis

The results of the real-time PCR analysis were processed using the 2^−∆∆CT^ method. Statistical analyses were performed using SPSS 22.0 software. The independent sample T-test and one-way analysis of variance (ANOVA), as well as LSD, Tukey’s-b, and Waller–Duncan tests, were used for variance analysis and significance tests. Data analysis was performed using GraphPad Prism 7.0 software. All experimental data are expressed as the mean ± standard error of the mean (SEM), and statistical significance was set at *p* < 0.05.

## 3. Results

### 3.1. HF Cycle Phases and FGF9 Expression in Relation to Sheep Wool Growth

**Figure 1:**
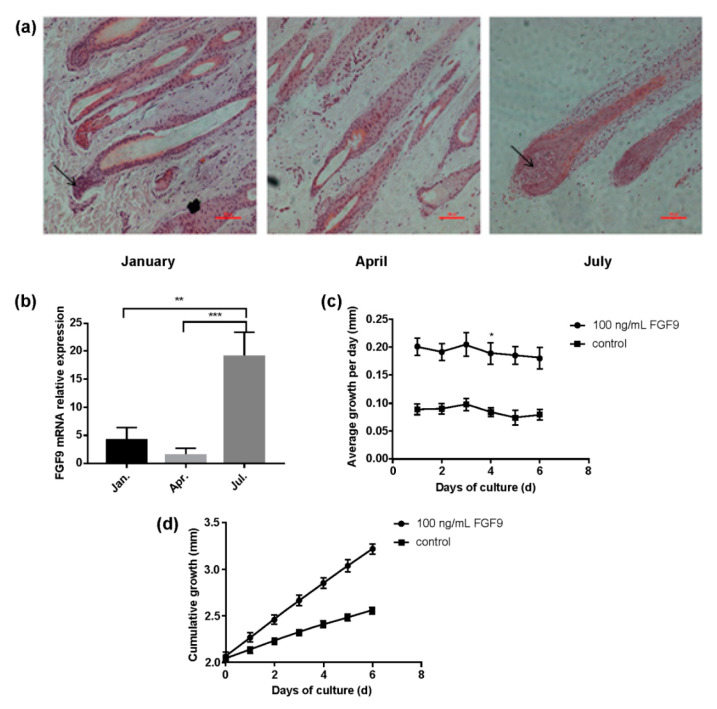
Involvement of *FGF9* in wool growth of small-tailed Han sheep. (**a**) Skin tissue sections of small-tailed Han sheep were collected at different periods of the hair follicle (HF) cycle. The arrow points to DP, which is not obvious in the telogen phases. (January: catagen phase; April: telogen phase; July: anagen phase). (**b**) Relative mRNA expression of *FGF9* in skin tissues at different periods (January (black): catagen phase; April (light grey): telogen phase; July (dark grey): anagen phase). (**c**) Comparison curves of the average daily growth of HFs cultured in a medium supplemented with 100 ng/mL *FGF9* protein (solid line with circles) and the control group (solid line with squares). (**d**) Comparison curves of the cumulative growth of cultured HFs in a medium supplemented with 100 ng/mL *FGF9* protein (solid line with circles) and the control group (solid line with squares). * *p* < 0.05; ** *p* < 0.01; *** *p* < 0.001.

The HF samples extracted in July 2022 were in the anagen phase, in which the DP consisted of fibroblasts embedded in the mesenchyme of connective tissues by the epithelial cells of the bulb. In January 2022, the HFs were in the catagen phase, in which the DPs were located at the proximal ends of the HFs and the DPCs were arranged closely together with minimal ECM. In April 2022, the HFs were in the telogen phase, in which the DP had reduced mesenchyme, the epithelial cells of the bulb shrank, and the DP was released (Figure 1a). These results confirm that the HFs in the skin of small-tailed Han sheep follow the HF cycle.

Furthermore, the *FGF9* gene is expressed in the skin tissues, HFs, and DPCs of small-tailed Han sheep (Supplementary Figure S1). The qPCR results show that *FGF9* expression changes throughout the HF cycle. Compared with that in the telogen phase where DP is released, the expression level of *FGF9* is higher in the skin tissues, where HFs are in the growth stage. It is speculated that the expression level of *FGF9* in the skin tissues may be related to the periodic growth cycle of hair follicles (Figure 1a,b). To evaluate the effects of *FGF9* on wool growth, HFs of small-tailed Han sheep were cultured in vitro using the organ culture method with the addition of the *FGF9* protein. Hair length markedly increases with the addition of *FGF9* protein compared to that in the control group (Figure 1c,d). These results demonstrate that *FGF9* participates in hair growth in small-tailed Han sheep.

### 3.2. FGF9 Promotes DPC Proliferation in Small-Tailed Han Sheep

**Figure 2:**
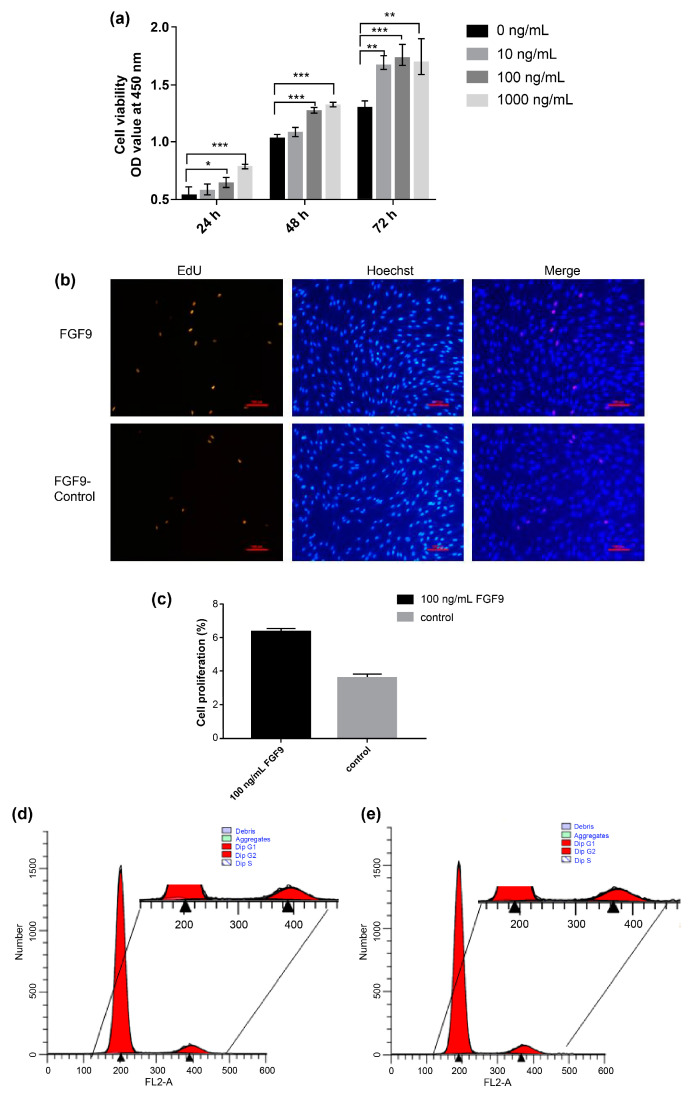
The *FGF9* protein promotes dermal papilla cell (DPC) proliferation. (**a**) Effects of different treatment durations and *FGF9* protein concentrations on DPC cell viability (0 ng/mL: solid line with circle; 10 ng/mL: solid line with square; 100 ng/mL: solid line with triangle; 1000 ng/mL: solid line with inverted triangle). (**b**) Evaluation of DPC proliferation using a 5-ethynyl-2 deoxyuridine (EdU) assay after treatment with 100 ng/mL *FGF9* protein for 48 h (EdU-positive cells: red, Hoechst 33,342 [nuclei]: blue). (**c**) The rate of cell proliferation (EdU-positive cells) as a percentage of the total number of cells. (**d,e**) Analysis of the DPC cycle after treatment with 100 ng/mL *FGF9* protein for 48 h (**c**: blank control group, **d**: 100 ng/mL *FGF9* treatment group). Red: Dip G1/2; white with blue lines: Dip S; light green: aggregates; blue: debris. * *p* < 0.05; ** *p* < 0.01; *** *p* < 0.001.

The CCK-8 assay results reveal that the addition of exogenous *FGF9* protein facilitates DPC proliferation (Figure 2a). We observed an increase in the number of EdU-positive cells in the proliferative phase following the addition of exogenous *FGF9* protein, and a statistical analysis of the number of EdU-positive cells as a proportion of the total number of cells does show an upward trend (Figure 2b,c). Cell cycle analysis shows that, compared to the control group, the number of cells in the S phase is higher after the addition of exogenous *FGF9* protein. In contrast, the number of cells in the G0/G1 phase is lower (Figure 2d,e), indicating that *FGF9* facilitates the cell cycle progression of DPCs.

**Figure 3:**
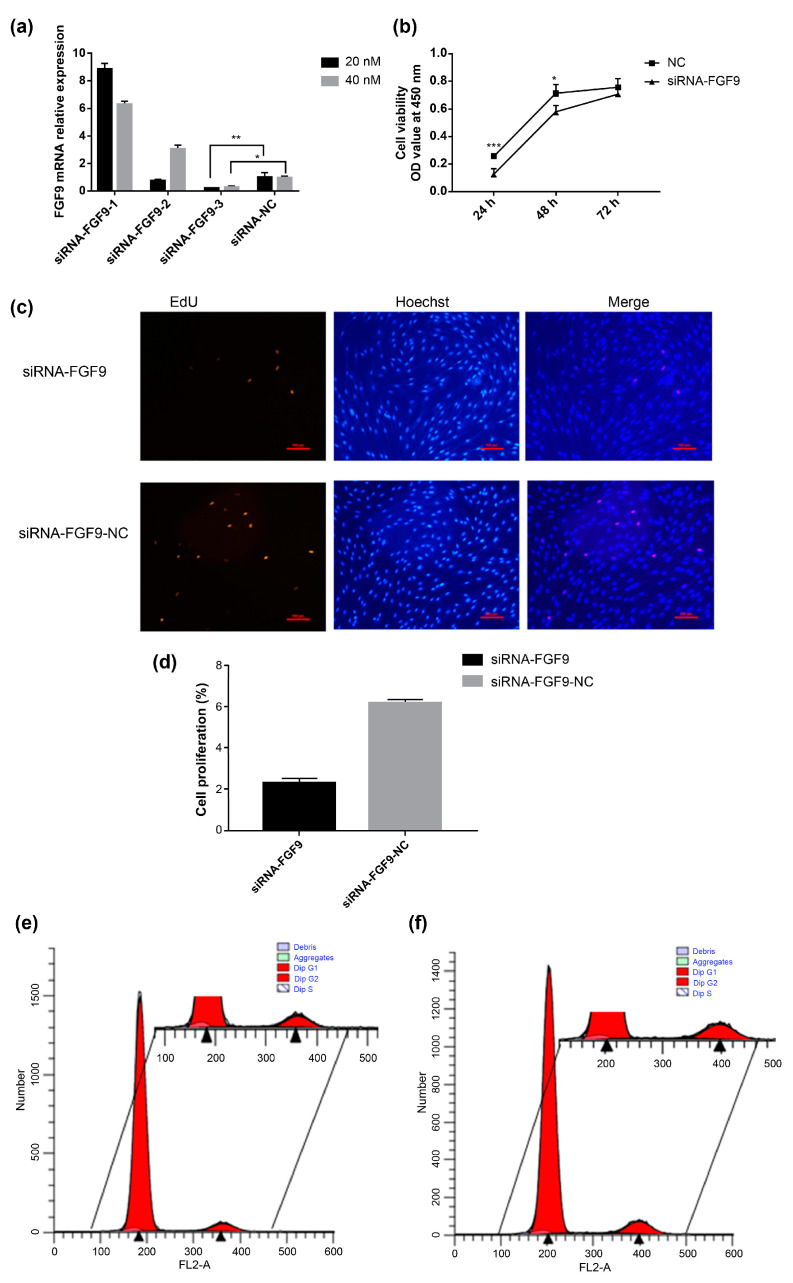
*FGF9* knockdown inhibits DPC proliferation. (**a**) Changes in *FGF9* expression levels after transfection with siRNA–*FGF9*-1, siRNA–*FGF9*-2, and siRNA–*FGF9*-3 for 48 h (black: 20 nM; grey: 10 nM). (**b**) Effects of different treatment durations on DPC proliferation after siRNA–*FGF9*-3 transfection; (negative control [NC]: solid line with squares; siRNA–*FGF9*: solid line with triangles). (**c**) Analysis of DPC proliferation using an EdU assay after 48 h of siRNA–*FGF9* transfection (EdU-positive cells: red, Hoechst33342 [nuclei]: blue. (**d**) The rate of cell proliferation (EdU-positive cells as a percentage of the total number of cells). (**e,f**) DPC cycle analysis after 48 h of siRNA–*FGF9* transfection (**e**: Cell transfection control [NC] group; **f**: siRNA–*FGF9*-transfected group). Red: Dip G1/2; white with blue lines: Dip S; light green: aggregates; blue: debris. * *p* < 0.05; ** *p* < 0.01; *** *p* < 0.001.

The qPCR results show that siRNA–*FGF9*-3 has the highest transfection efficiency at 20 nM (Figure 3a). Dermal cell papilla at the growth stage are transfected with siRNA–*FGF9*-3 (hereinafter called siRNA–*FGF9*). The CCK-8 assay results show that cell proliferation is reduced after the inhibition of *FGF9* expression compared with that in the control group (Figure 3b). The EdU assay results reveal a decrease in the number of positive DPCs in the proliferation phase after *FGF9* expression inhibition, and statistical analysis of the number of EdU-positive cells as a proportion of the total number of cells does show a decreasing trend (Figure 3c,d). Cell cycle assay results show a significant decrease in the number of cells in the S phase after *FGF9* inhibition and an increase in the number of cells in the G0/G1 phase compared to those in the control (Figure 3e,f).

### 3.3. FGF9 Regulates the Expression of Key Wnt/β-Catenin Signaling Pathway Genes

**Figure 4:**
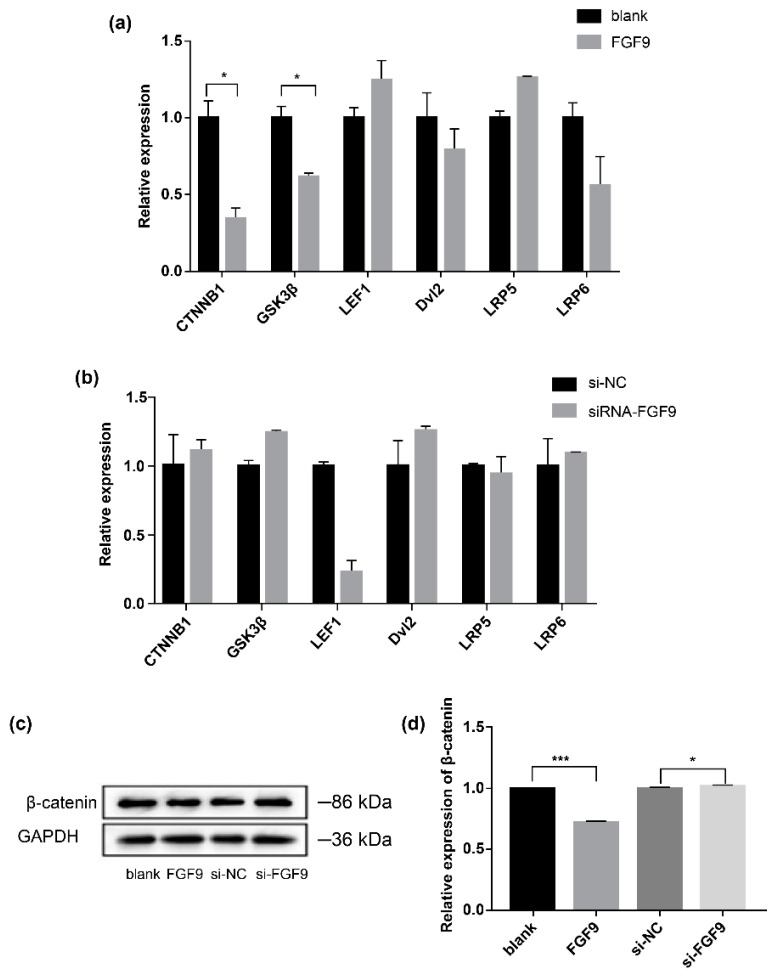
*FGF9* regulates the expression of key Wnt/β-catenin signaling pathway genes. (**a**) Effects of *FGF9* protein supplementation on the expression of key Wnt/β-catenin signaling pathway genes (black: blank; grey: *FGF9*) in DPCs. (**b**) Effects of *FGF9* expression inhibition on the expression of key Wnt/β-catenin signaling pathway genes (black: si-NC; grey: siRNA–*FGF9*) in DPCs. (**c**) Western blot showing the effects of *FGF9* protein supplementation and *FGF9* knockdown on the protein expression of β-catenin, a marker protein of the Wnt/β-catenin signaling pathway. (**d**) Greyscale value analysis of the changes in β-catenin expression (black: blank; medium grey: *FGF9*; dark grey: si–NC; light grey: si–*FGF9*). * *p* < 0.05; *** *p* < 0.001.

We examined the effects of *FGF9* on the mRNA expression of key genes of the Wnt/β-catenin signaling pathway, including *CTNNB1*, *GSK3β*, *LEF1*, *DVL2*, *LRP5*, and *LRP6*, as well as that of *β-catenin*, a marker protein of the Wnt/β-catenin signaling pathway. After the exogenous addition of *FGF9* protein to DPCs, the mRNA expression levels of *CTNNB1*, *GSK3β*, *DVL2*, and *LRP6* decrease, with a significant decrease observed in that of *CTNNB1* and *GSK3β*, whereas the expression levels of *LEF1* and *LRP5* increase (Figure 4a). Although there is no significant difference between them, compared with the control group, the mRNA expressions of *CTNNB1*, *GSK3β*, *DVL2,* and *LRP6* in the *FGF9* inhibited group show an upregulated trend, while the mRNA expressions of *LEF1* and *LRP5* show a downward trend (Figure 4b). Western blotting shows similar results; the protein expression levels of β-catenin decrease after the addition of *FGF9* protein but increase after the inhibition of *FGF9* expression (Figure 4c,d). Our findings show that the proliferation-promoting effects of *FGF9* on DPCs may be regulated via the Wnt/β-catenin signaling pathway.

### 3.4. Functional Enrichment Analysis of DEGs

**Figure 5:**
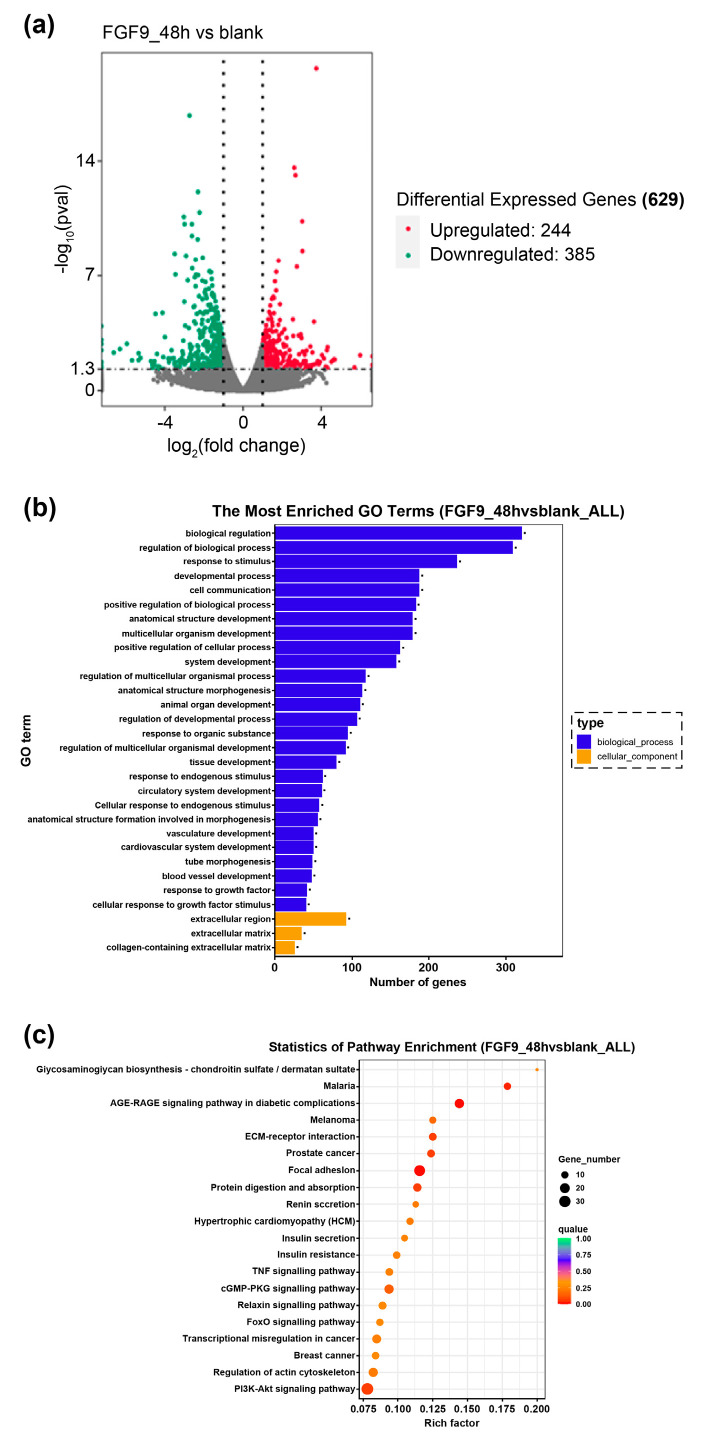
(**a**) Volcano plots of differential gene expression between DPCs treated with *FGF9* for 48 h and the blank group (green: downregulated genes; red: upregulated genes). (**b**) Histogram of Gene Ontology (GO) enrichment in the biological processes (blue) and cellular composition (yellow) categories. The significance level of the enrichment is set to an adjusted *p*−value (*p*_adjust_ < 0.05). (**c**) Scatterplot for the Kyoto Encyclopedia of Genes and Genomes (KEGG) enrichment of differential genes. The bigger the circle, the more genes there are. The redder the colour, the smaller the Q−value, and the closer the Q−value number is to zero, the more it is significantly enriched.

To gain a comprehensive understanding of how *FGF9* regulates DPC proliferation, we performed transcriptome analysis on DPCs in the *FGF9* protein-supplemented and control groups. The changes in the differential expression of genes between the two groups were analysed using a volcano plot. A total of 629 DEGs are identified, of which 385 genes are downregulated and 244 genes are upregulated (Figure 5a).

From the KEGG analysis, most single genes are associated with biological regulation and developmental processes. In the cellular components category, most single genes are associated with the ECM. The top 30 most significant GO terms are not enriched in the molecular function category (Figure 5b). The KEGG pathway enrichment analysis was then performed on the significant DEGs screened from the *FGF9*-treated and control groups, with the most enriched category being glycosaminoglycan biosynthesis-chondroitin sulphate/dermatan sulphate, followed by malaria, the roles of advanced glycation end-product (AGE-RAGE) signaling pathways in diabetic complications, and ECM receptor interactions. The top 20 most significant KEGG pathways were selected in the order of their *p*-values and plotted (Figure 5c).

## 4. Discussion

The results indicate that *FGF9* can accelerate the proliferation and cell cycle of DPCs and regulate HF growth and development through the Wnt/β-catenin signaling pathway. *FGF9* expression also varies throughout the HF cycle and participates in wool growth in small-tailed Han sheep. Similarly, previous studies indicate that FGF5, which also belongs to the FGF family, can affect the density of sheep wool by regulating the Wnt/β-catenin signaling pathway [32].

Sectioned skin tissues of small-tailed Han sheep are found to follow the HF cycle, showing regular anagen, catagen, and telogen phases. Additionally, the expression levels of *FGF9* varies according to the HF cycle. Similarly, the expression of *FGF7*, *FGF5*, *FGF10*, and *FGF22* is also found to peak during the anagen phase in mice [16]. The cell proliferation rate and cell cycle are accelerated in DPCs treated with exogenous *FGF9* protein. Simultaneously, the proliferation rate and cell cycle are inhibited in *FGF9*-downregulated DPCs. This is consistent with the results of Biggs et al. [33] and Gay et al. [19], who show that *FGF9* promotes cellular proliferation and HF regeneration. The results of the in vitro HF culture confirm the facilitating effect of *FGF9* on hair shaft elongation. Likewise, Cai et al. [34] applied oil produced by *FGF9*-transgenic safflower seeds to the skin and wounds of mice and found that the hair growth and wound healing rates were higher in the treatment group than those in the control group.

Some previous studies partially explained the association between FGF family members and mechanisms related to the Wnt/β-catenin signaling pathway. Notably, FGFs and the Wnt signaling pathway interact within the DP to regulate the expression of various molecules, including Wnt agonists (R-spondins) and antagonists (DKK2 and Notum) [35]. β-catenin deficiency leads to the degeneration and inflammation of HFs [36]. However, a forced expression of the constitutively active form of β-catenin in the DP does not impair the hair cycle [37]. These results suggest that, while Wnt signaling activity in the DP is essential for maintaining growth, it is not sufficient to counteract hair-promoter-induced signals. Herein, we found that the mRNA and protein expression of a marker gene of the Wnt/β-catenin signaling pathway, *CTNNB1*, is significantly lower (*p* < 0.05) in DPCs supplemented with *FGF9* protein compared to the control group, while it shows an upregulation trend in DPCs with *FGF9* downregulation. Therefore, *FGF9* may regulate the growth and development of HFs through the Wnt/β-catenin signaling pathway. Similarly, Enshell-Seijffers et al. [38] show that β-catenin activity in the DP regulates signaling pathways, including that of FGF, thereby mediating the inducing effects of the DP. In conjunction with subsequent experiments, we hypothesised that the positive effect of *FGF9* on the proliferation of DPCs may be synergistic through multiple signaling pathways. The signaling cascade in which *FGF9* acts in DPCs may act antagonistically with Wnt/β-catenin, and competitive inhibition leads to a decrease in *CTNNB1* expression.

The synergistic involvement of multiple signaling pathways in DPC proliferation has been established, and studies show that FGF signaling crosses and acts in concert with the NOTCH, WNT, and TGF-β signaling pathways to control cell maturation [12,13]. Owing to the insignificant changes in the mRNA expression of *LEF1*, *DVL2*, *LRP5*, and *LRP6*, we speculated that *FGF9* might regulate DPC proliferation through other signaling pathways. Therefore, we performed transcriptomic analysis of DPCs in the *FGF9* protein-treated and control groups. Through GO and KEGG pathway enrichment analyses of the DEGs, we identified the genes most associated with *FGF9* expression that regulate the biological processes and cellular composition during DPC proliferation. In the cellular composition category, most single genes are enriched in the ECM, which is consistent with the fact that *FGF9* exerts its biological functions primarily through a paracrine mechanism of action [39]. In the biological processes category, the DEGS are involved in the regulation of biological and developmental processes. Several DEGs are enriched in various signaling pathways, such as TNF, cGMP-PKG, relaxin, FoxO, and PI3K-Akt, suggesting that *FGF9* may play a role in promoting cell cycle transition and regulating cell metabolism. By analysing the sequencing results, we speculate that *FGF9* synergistically regulates DPC proliferation through multiple signaling pathways. However, the mechanisms of interaction between *FGF9* and various signaling pathways still need to be further explored.

In conclusion, our study shows that *FGF9* is expressed in varying extents in small-tailed Han sheep and that the addition of exogenous *FGF9* protein can encourage DPC proliferation and cell cycle progression. Furthermore, *FGF9* may regulate HF growth and development through the Wnt/β-catenin signaling pathway. Lastly, the positive effects of *FGF9* on DPC proliferation and the cell cycle may be the result of the synergistic effects of multiple signaling pathways. These findings provide the basis for further production of genetically modified sheep that produce more and better wool. Future research will focus on the molecular mechanism through which *FGF9* promotes DPC proliferation to elucidate how *FGF9* induces hair follicle growth and development through multiple signaling pathways.

## Figures and Tables

**Table 1:** Small interfering RNA (siRNA)–fibroblast growth factor 9 (*FGF9*) sequences used in this study.

Name	Sequences
siRNA–*FGF9*-1	sense: 5′-GCAGGACUGGAUUUCACUUTT-3′
	anti-sense: 5′-AAGUGAAAUCCAGUCCUGCTT-3′
siRNA–*FGF9*-2	sense: 5′-GACUCUACCUCGGCAUGAATT-3′
	anti-sense: 5′-UUCAUGCCGAGGUAGAGUCTT-3′
siRNA–*FGF9*-3	sense: 5′-GCACCAGAAAUUCACCCAUTT-3′
	anti-sense: 5′-AUGGGUGAAUUUCUGGUGCTT-3′

**Table 2:** Primer sequences used for qPCR analysis.

Gene Name	Sequences (5′→3′) (F: Forward, R: Reverse)	Length (bp)	Tm (°C)
*FGF9-C*	F: TACGGTCGGATGAGATGAA	1070	60
	R: TTGGCAACAATGGATGTG		
*FGF9-R*	F: AGCCAAAGTTGACAAAGACCGTT	92	60
	R: AGCAAATCAATAGGGACCCACCG		
*CTNNB1*	F: GGCTACTGTTGGGTTGATTCG	215	60
	R: GGATGTGAAGGGCTCCAGTA		
*GSK3β*	F: ATAATCAAGGTCCTGGGAACAC	200	60
	R: TCCAGCAGACGGCTACAAA		
*LEF1*	F: GCATCCCTCATCCAGCCATC	112	60
	R: GGCTCCTGCTCCTTTCTCTG		
*DVL2*	F: TACCTGGTGAAGATCCCCGT	262	60
	R: GTGGAGGAGCAAGGTCTGTC		
*LRP5*	F: CATCGTGGACTCTGACATTTACTG	228	60
	R: TTACAGGCGTGGATGGAGC		
*LRP6*	F: TGATCTTTCAGGGGCCAACC	73	60
	R: AAACACAGTCAGTCCCACAGG		
*ACTB*	F: CCGCAAATGCTTCTAGGCGG	98	60
	R: TCGCACGAGGCCAATCTCAT		

## Data Availability

The data presented in this study are available upon request from the corresponding author.

## Supplementary Materials

The following supporting information can be downloaded at: https://www.mdpi.com/article/10.3390/genes14051106/s1, Figure S1: The *FGF9* gene is widely expressed in the skin of small-tailed Han sheep.

## Abbreviations

Hair follicles: HFs; dermal papillae: DP; dermal papilla cells: DPCs; insulin-like growth factor 1: IGF-1; keratinocyte growth factor: KGF; hepatocyte growth factor: HGF; fibroblast growth factor: FGF; fibroblast growth factor receptor: FGFR; notch homologue protein: NOTCH; transforming growth factor: TGF; bovine serum albumin: BSA; phosphate-buffered saline: PBS; small interfering RNA: siRNA; cell counting kit-8: CCK-8; catenin **β** 1: *CTNNB1*; glycogen synthase kinase-3β: GSK3β; lymphoid enhancer binding factor 1: LEF1; dishevelled segment polarity protein 2: *DVL2*; low-density lipoprotein (LDL) receptor-related protein: LRP; complementary DNA: cDNA; polyvinylidene difluoride: PVDF; enhanced chemiluminescence: ECL; extracellular matrix: ECM; Dickkopf-related protein 2: *DKK2*; tumour necrosis factor: TNF; cyclic guanosine monophosphate–protein kinase G: cGMP-PKG; forkhead box O: FoxO; phosphoinositide-3-kinase–protein kinase B: PI3K-Akt.
