# Supplementary figures and images for: Regulatory Effects of FGF9 on Dermal Papilla Cell Proliferation in Small-Tailed Han Sheep
Source: Genes (Basel). 2023 May 18;14(5):1106. doi: 10.3390/genes14051106 (PMC10218283; doi:10.3390/genes14051106)

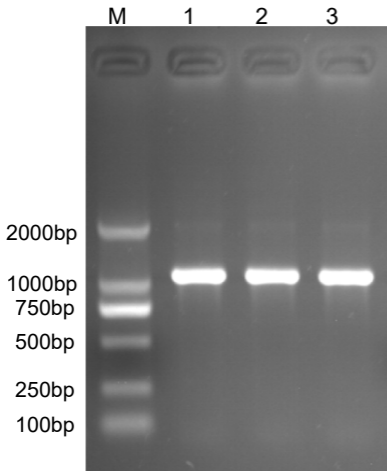

M: DL2000 marker; 1: skin tissue; 2: hair follicles; 3: DPC.

Figure 1. RT PCR results of FGF9 gene in sheep skin tissue

Supplement: Supplementary file 1 [file genes-14-01106-s001.zip › Supplement.pdf]
